# The effect of perceived stress on binge eating behavior among Chinese university students: a moderated mediation model

**DOI:** 10.3389/fpsyt.2024.1351116

**Published:** 2024-03-18

**Authors:** Chunlu Li, Jing Gu, Yixin Li, Baijuan Xia, Xiaolu Meng

**Affiliations:** ^1^ Department of Histology and Embryology, School of Basic Medical Sciences, Guizhou Medical University, Guiyang, China; ^2^ Key Laboratory for Research on Autoimmune Diseases of Higher Education schools in Guizhou Province, Guiyang, China; ^3^ Department of Psychology, School of Medical Humanitarians, Guizhou Medical University, Guiyang, China; ^4^ Guizhou Health Development Research Center, Guiyang, China

**Keywords:** perceived stress, life history strategy, binge eating behavior, distress tolerance, Chinese university students

## Abstract

**Introduction:**

Previous studies have demonstrated a strong link between perceived stress and binge eating behavior, but the psychological mechanisms underlying such phenomenon are not fully understood. The present study further addressed this issue in a life history framework, focusing on life history strategy and distress tolerance.

**Methods:**

Firstly, we investigated the mediation role of life history strategy on the relationship between perceived stress and binge eating behavior. Secondly, we examined the moderation role of distress tolerance on the effect of perceived stress on life history strategy, as well as on the direct effect of perceived stress on binge eating behavior. We analyzed data from 1342 Chinese university students.

**Results:**

Results indicated that life history strategy mediates the relationship between perceived stress and binge eating behavior; distress tolerance has significant moderating effects on the direct effect of perceived stress on binge eating behavior and their indirect effect via life history strategy.

**Discussion:**

Therefore, distress tolerance skills training and life history-based interventions might be potentially effective ways to reduce binge eating behavior triggered by perceived stress.

## Introduction

Binge eating is characterized by rapid and excessive food consumption, often accompanied by a feeling of loss of control (1). From 2018 to 2020, the global prevalence of binge eating is estimated to be 0.6-1.8% among adult women and 0.3-0.7% among adult men (2). A study in China found 85 cases of binge eating behavior among 1,103 college students based on the scores of the binge eating scale (3). Binge eating often leads to poor personal quality of life, work, family (4), because of its negative consequences including non-suicidal self-injury (4), gastric perforation and acute gastric dilatation (5). Given its pervasiveness and serious negative consequences, binge eating has become a significant public health concern.

Laboratory, descriptive, and ecological momentary assessment studies have demonstrated that binge eating is preceded by negative affect accompanying perceived stress (6), and that perceived stress positively predicts binge eating behavior (7, 8). Further, individuals with binge eating behavior have higher level of perceived stress and negative emotional experience than healthy controls under the same stressful laboratory situation (9). However, the psychological mechanisms by which perceived stress leads to binge eating are still not fully understood.

Existing researches have mainly addressed this issue from the perspective of self-regulation or coping strategies. For example, higher level of psychological distress (including stress) and emotional eating was observed in individuals with emotional dysregulation, an important aspect of self-regulation (10). Meanwhile, more coping strategies (including both positive and negative coping strategies) to relieve stress and more catastrophizing were reported by individuals with binge eating in stressful tasks (11). The risk of binge eating was increased by distraction coping increases, while decreased by social support (7). More directly, the relationship between perceived stress and binge eating/emotional eating is mediated by eating self-regulation (12) and emotion-focused coping (13) and moderated by resilience (14), cognitive reappraisal, but not response inhibition (15). In this research perspective, binge eating behavior is regarded as maladaptive behavior because of its negative consequences in the long run.

On the other hand, binge eating also is proposed to be motivated by a desire to escape from self-awareness, it might be an emotion regulation strategy to avoid unpleasant emotional distress accompanying aversive self-perceptions (16). Specifically, binge eating is a concrete and palatable way to distract from concerns about emotional distress. It seems that the expectation that binge eating will help to alleviate negative affect leads to the occurrence of this behavior (17–19). Therefore, binge eating appears to be a proactive adaptation strategy effective at the moment. In this context, it may be an adaptive behavior for binge eaters under special circumstances where due to excessive pressure, they have to focus on the present and cannot take into consideration the future. That is a fast life history strategy according to life history theory in evolutionary psychology.

Life history theory has been developed as an explanation for differences in energy and time allocation patterns between and within species (20). Resources are limited for all living organisms. How to allocate their limited resources is critical to the survival and continued existence of the species. The strategies for allocating resources often change based on their assessment of environmental stress. In predictable environments, planning and working for higher future rewards is cost effective. Therefore, cognition and behavior of humans and animals tend to be more future oriented than present oriented. That is to say, they prefer to behave in ways that are likely to be rewarded in the future but little or no immediate benefit. This strategy is called the slow life history strategy. Conversely, when the future is uncertain and hard to be predicted, it is unlikely that investing will pay off in the future. Therefore, a fast strategy is more adaptive, with organisms focusing more on the present and discounting the future (21).

Environmental stress is key to inducing a fast life history strategy (22). In the human life history strategy, more emphasis is placed on individual’s subjective perception in term of environmental stress (23, 24). That is to say, perceived stress, rather than objective environmental stress, more accurately reflects an individual’s stress state and more effectively triggers a fast life history strategy. Individuals with binge eating behavior suffer from high standards and expectations, particularly an acute sensitivity to the difficult (perceived) demands of others (16); This psychological trait exacerbates the state of imbalance between environmental demands and the individual’s capacity to cope with these demands, resulting in more perceived stress. In fact, they have higher levels of perceived stress under the same stressful situation (9). Therefore, they are expected to have a faster life history strategy. Further, a number of reviews or studies have examined a variety of psychopathological symptoms within a life history strategy perspective (17, 25), including eating disorders (26, 27). In contrast, slow life history strategy has a function in protecting against disordered eating behaviors (28). Based on these evidences, it is reasonable to assume that fast life history strategy may mediate the relationship between perceived stress and binge eating (H1).

Distress tolerance (DT) refers to the perceived and actual capability to withstand aversive physical and psychological states (29, 30), including uncertainty, ambiguity, frustration, negative emotion, physical discomfort (31). Individuals with lower DT are thought to be more reactive to distress, more likely to experience stress overload (32) and more attempt to avoid these aversive states (29). That is to say, under the same perceived stressful situation, individuals with low distress tolerance are more likely to experience psychological overload followed by a stronger motivation to alleviate negative experiences, finally results in the initiation of the fast life history strategy and occurrence of binge eating behavior. Therefore, it seems reasonable to hypothesize that distress tolerance moderates the relationship between perceived stress and fast life history strategy (H2) and that distress tolerance moderates the relationship between perceived stress and binge eating (H3).

**Figure 1:**
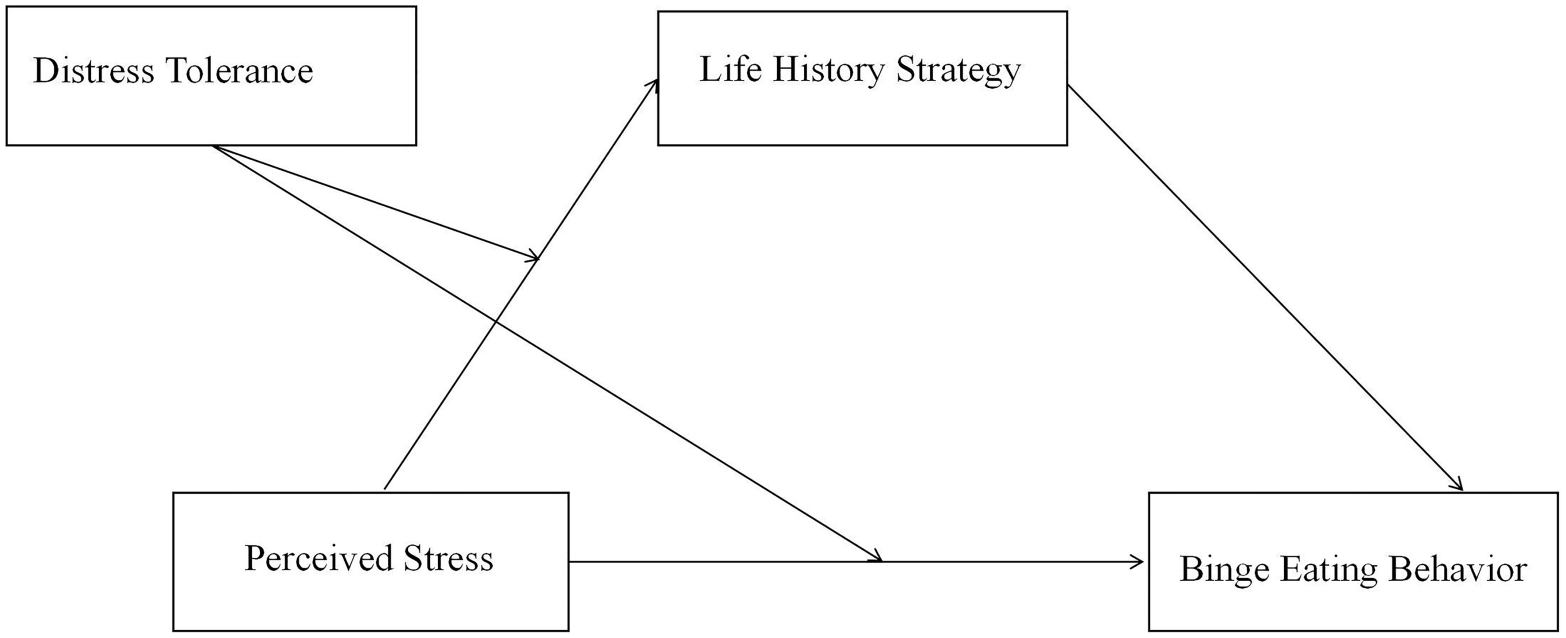
The moderated mediation model hypothesized in the present study. In this model, we hypothesized that fast life history strategy may mediate the relationship between perceived stress and binge eating (H1); and that distress tolerance may moderate both the relationship of perceived stress and fast life history strategies (H2) and that of perceived stress and binge eating (H3).

Together, the present study aimed to investigate the psychological mechanism underling the role of perceived stress in the occurrence of binge eating behavior in Chinese culture. First, we tested whether life history strategy independently mediates their relationship. Then, we investigated whether distress tolerance moderates the direct effect of perceived stress on binge eating behavior and/the life history strategies. The hypothetical model was shown in **Figure 1**.

## Materials and methods

### Participants

This research was approved by the ethics committee of Guizhou Medical University. Chinese undergraduate students from a university in Guizhou province were recruited. Prior to participation, all students were asked to sign informed consent forms and informed that their responses would be anonymous with the option of withdrawal at any time. Data of the subjects who chose the same answer for more than half of the total scale were excluded (33). Finally,1342 students (519 males and 823 females) aged 18-24 (M = 19.03, SD = 1.02) were included in data analysis.

### Measures

#### Binge eating behavior

To measure binge eating behavior, the Chinese version of Binge Eating Scale (BES) adapted by Wu Siyao, et al. (34) was adopted. The scale consists of 16 items, each with 3 to 4 options. The total score of the scale ranges from 0 to 46 points, higher total score indicates a more severe level of binge eating behavior. In this study, the Cronbach’s alpha was 0.79.

#### Perceived stress

To measure perceived Stress, the Chinese version of Perceived Stress Scale (PSS) revised by Tingzhong Yang (35) was adopted. PSS is developed by Cohen et al. (36) and includes two dimensions: tension (e.g, Can’t cope with all the tasks on your own.) and sense of loss of control (e.g., Things are going as hoped.). Each dimension contains 7 items, using a 5-Likert point scale. Items belonging to the sense of loss of control dimension were scored in reverse. The total score is from 0 to 56, with higher score indicating higher perceived stress. In this study, the Cronbach’s alpha was 0.77.

#### Life history strategy

To measure life history strategies, the life history strategy Scale revised by Sai Xueying et al. (37) was adopted. There are 20 items in the original scale and using a 7-Likert point scale. It’ s important to mention that item 20”I believe in religion and regularly participate in religious activities” has been removed in the Chinese version of the Life History Strategy Scale (37). The total score is derived from the summation of individual scores. Higher score suggests propensity towards slow life history strategy, whereas lower score indicates preference for fast life history strategy. In this study, the Cronbach’s alpha was 0.88.

#### Distress tolerance

To measure Distress tolerance, the Distress Tolerance Scale (DTS) the revised by You Jianing et al. (38) was adopted. The scale consists of 16 items, which was scored on a 5-Likert point scale. The scale has a total score range of 15-75, where higher DTS score indicates lower distress tolerance. In this study, the Cronbach’s alpha was 0.83.

### Statistical analysis

The data gathered in this study were analyzed as outlined by Hayes (39) in his book. SPSS 26.0 was used to conduct common method bias test, descriptive statistical analysis and correlation analysis of the data. Then, the mediation effect of life history strategies was examined by PROCESS macro model 4 and the moderation role of distress tolerance was examined by model 1. Finally, all the study hypotheses were tested simultaneously by model 8. Data were calculated based on standardized scores. Bootstrapped confidence interval (CI; 5,000 bootstrap samples) for the indirect effect was obtained. If zero is not included in the confidence interval, effects are significant.

## Results

### Common method biases tests

To exclude common method bias, the Harman single factor method was used in this study (40). The results showed that there were 14 factors with feature roots greater than 1, among which the cumulative variance explained by the first factor was only 16.918%, less than the critical value of 40% (41). Therefore, common method biases are unlikely to confuse the interpretation of the data analysis.

### Describe statistics and correlation analysis

**Table 1** shows the descriptive statistics and correlations of the variables in this study. Perceived stress was negatively correlated with life history strategy (*r* = - 0.432, *p* < 0.001) and positively correlated with binge eating behavior (*r* = 0.362, *p* < 0.001). Life history strategy was negatively correlated with binge eating behavior (*r* = -0.253, *p* < 0.001). Distress tolerance was positively correlated with perceived stress (*r* = 0.565, *p* < 0.001) and negatively correlated with life history strategy (*r* = - 0.28, *p* < 0.001), while positively correlated with binge eating behavior (*r* = 0.368, *p* < 0.001).

**Table 1:** Descriptive statistics and correlation of variables.

Variable	*M ± SD*	1	2	3	4
1. Perceived Stress	24.33 ± 6.29	1			
2. Life History Strategy	102.21 ± 14.43	-0.432^***^	1		
3. Binge Eating Behavior	7.91 ± 5.87	0.362^***^	-0.253^***^	1	
4. Distress Tolerance	37.94 ± 8.77	0.565^***^	-0.28^***^	0.368^***^	1

N=1342, ^***^ p < 0.001.

### Testing for mediation effect

To test hypothesis 1, PROCESS macro-Model 4 was used. The specifications of this model can be seen in **Table 2**. Since Gender and BMI are important variables that affect eating behavior and may confound the interpretation of our results (42–44), they were selected as covariates in the present study. After controlling for gender and BMI, the mediator and dependent variable models showed that perceived stress was negatively correlated with life history strategy (*β* = -0.73, *p* < 0.001. see Model 1 of **Table 2**), and life history strategy was negatively correlated with binge eating behavior (*β* = -0.06, *p* < 0.001. see Model 2 of **Table 2**). While, perceived stress was also positively associated with binge eating behavior (*β* = 0.25, *p* < 0.001. see Model 2 of **Table 2**). Therefore, life history strategy partly mediated the relationship between perceived stress and binge eating behavior, supporting Hypothesis 1.

**Table 2:** Testing the mediation effect of Life History Strategy on binge eating behavior.

Model 1 (Life History Strategy)					Model 2 (Binge Eating Behavior)			
Constant	*β*	*t*	*SE*	95% CI	*β*	*t*	*SE*	95% CI
Gender	0.16	4.06^***^	0.04	0.08 ~ 0.23	0.17	8.80^***^	0.02	0.13 ~ 0.20
BMI	0.00	0.24	0.01	-0.01 ~ 0.01	0.01	3.71^***^	0.00	0.01 ~ 0.02
PS	-0.73	-17.50^***^	0.04	-0.81 ~ -0.65	0.25	11.09^***^	0.02	0.20 ~ 0.29
LHS					-0.06	-4.22^***^	0.00	-0.08 ~ -0.03
R^2^	0.19				0.19			
F	106.27				77.62			

N=1342, the Beta value are standardized coefficients, thus they can be compared to determine the relative strength of different variables in the model. Each column is a regression model that predicts the criterion at the top of the column.PS, perceived stress, LHS, life history strategy. ^*^p<0.05, ^***^p<0.001.

### Testing for the moderation effect

To examine the moderation effect of distress tolerance on the relationship between perceived stress and life history strategy, the PROCESS macro model 1 was used. Results was shown in **Table 3**. The interaction between perceived stress and distress tolerance on life history strategy was significant (*β* = 0.14, *p* < 0.05; see Model 3 of **Table 3**). Thus, distress tolerance had a moderating role between perceived stress and life history strategy, supporting Hypothesis 2. Similarly, PROCESS macro model 1 was also ran to test the moderation effect of distress tolerance on the relationship between perceived stress and binge eating behavior. Results was also shown in **Table 3**. The interaction between perceived stress and distress tolerance on binge eating behavior was also significant (*β* = 0.06, *p* < 0.05. see Model 4 of **Table 3**), supporting Hypothesis 3.

**Table 3:** Testing the moderation effect of distress tolerance.

Model 3 (Life History Strategy)					Model 4 (Binge Eating Behavior)			
Constant	*β*	*t*	*SE*	95%CI	*β*	*t*	*SE*	95%CI
Gender	0.17	4.26^***^	0.04	0.09 ~ 0.24	0.14	7.68^***^	0.02	0.11 ~ 0.18
BMI	0.00	0.16	0.01	-0.01 ~ 0.01	0.01	3.99^***^	0.00	0.01 ~ 0.02
PS	-1.01	-6.51^***^	0.16	-1.32 ~ -0.71	0.18	7.68^***^	0.02	0.14 ~ 0.23
DT	-0.31	-2.75^*^	0.11	-0.54 ~ -0.09	0.14	7.73^***^	0.02	0.11 ~ 0.18
PS×DT	0.14	2.28^*^	0.06	0.02 ~ 0.25	0.06	2.16^*^	0.03	0.01 ~ 0.12
R^2^	0.20				0.22			
F	65.73				74.17			

N=1342, The Beta value are standardized coefficients, thus they can be compared to determine the relative strength of different variables in the model. Each column is a regression model that predicts the criterion at the top of the column.PS, perceived stress, DT, distress tolerance. *p<0.05, ***p<0.001.

**Figure 2:**
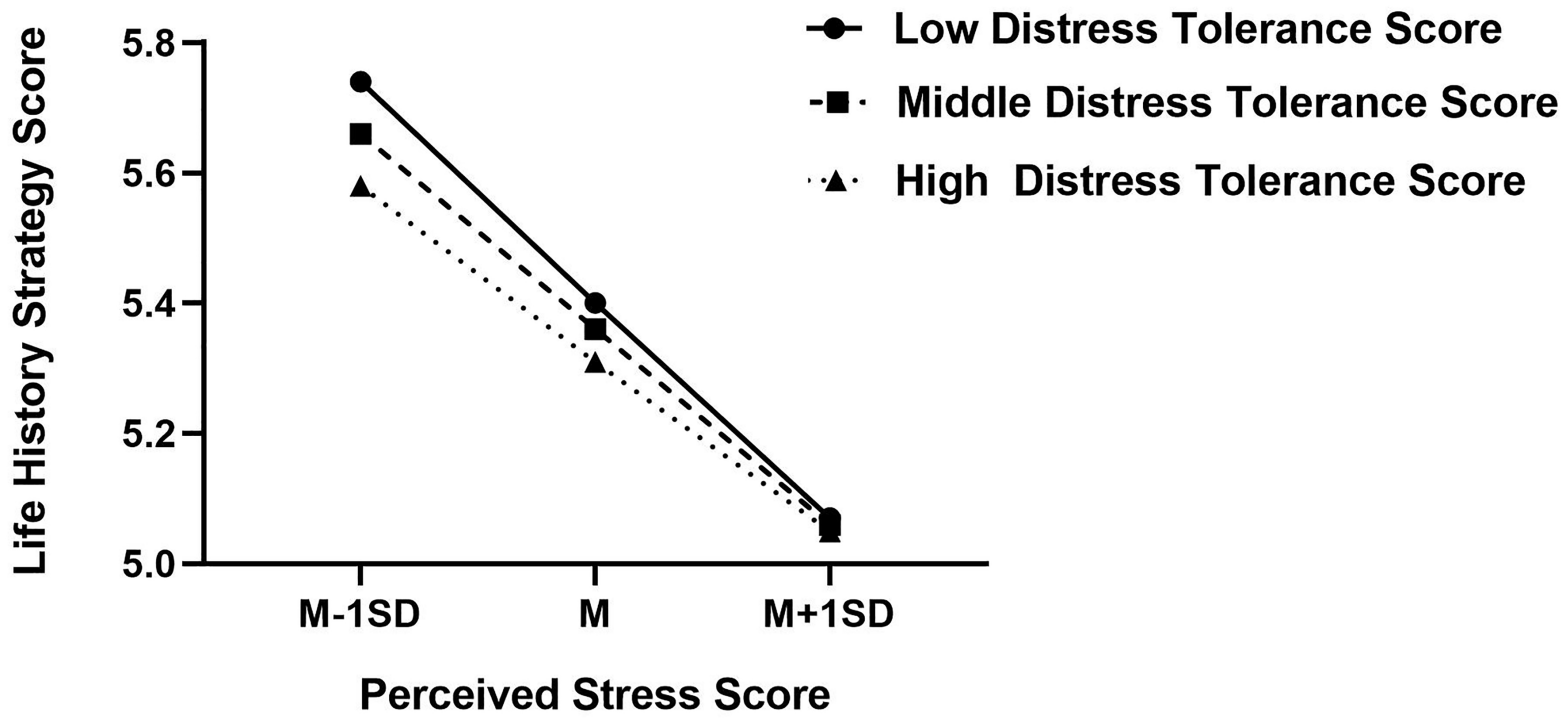
Interaction effect of distress tolerance and perceived stress on life history strategy. High and low levels of perceived stress and distress tolerance represent one standard deviation above and below the mean, respectively. Higher life history strategy score indicates a tendency to use slower life history strategy, and conversely, lower score indicates a tendency to use faster life history strategy. Higher distress tolerance score indicates lower distress tolerance; in contrast, lower distress tolerance score indicates higher distress tolerance.

To describe it more directly, figure of predicted perceived stress against life history strategy was plotted in **Figure 2**. Low and high level of distress tolerance score (1- SD below the mean and 1+ SD above the mean, respectively) were showed in the figure separately. The results showed that when perceived stress is high, both individuals with higher distress tolerance and those with lower distress tolerance tend to use the fast life history strategy; while perceived stress was low, individuals with low distress tolerance were more inclined to use faster life history strategy than those with high distress tolerance.

**Figure 3:**
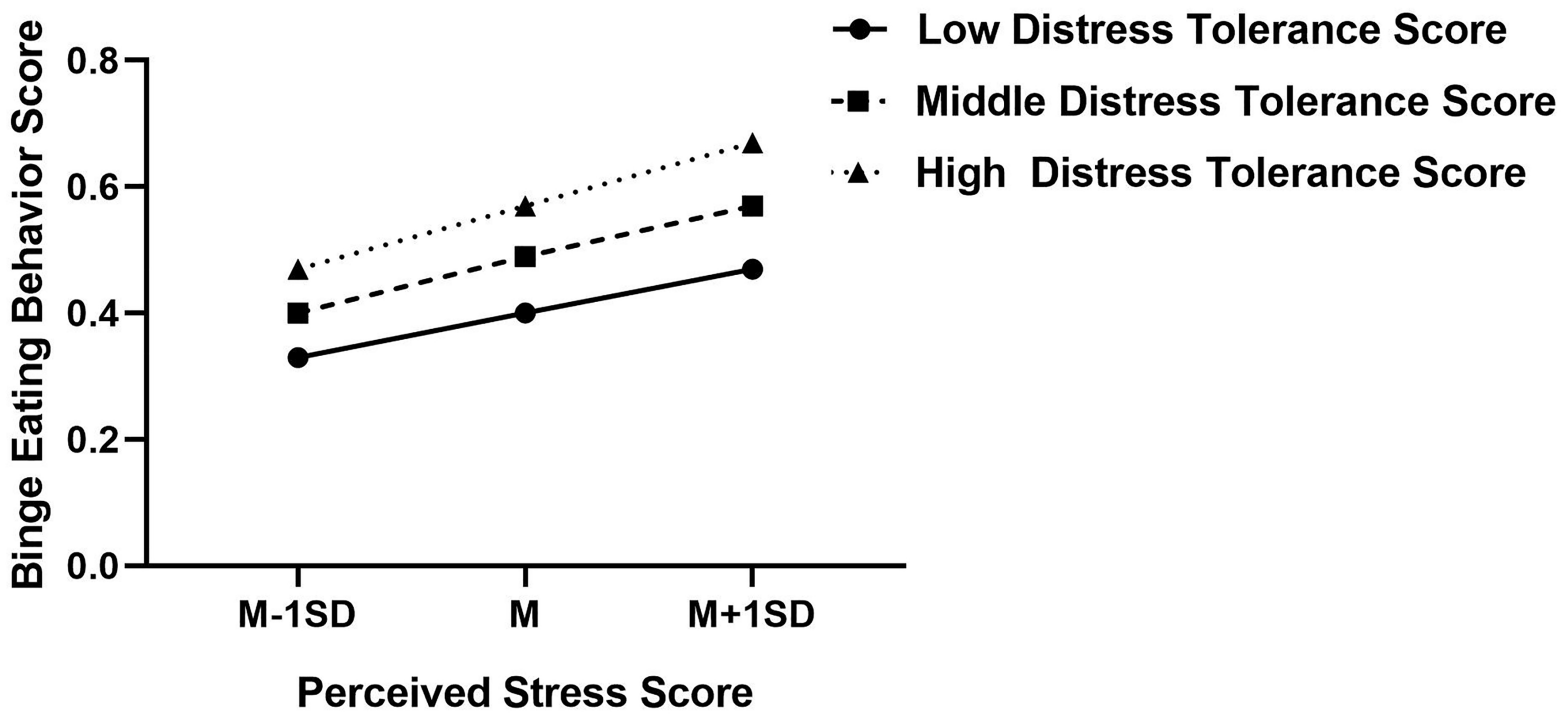
Interaction effect of distress tolerance and perceived stress on binge eating behavior. High and low level of perceived stress and distress tolerance represent one standard deviation above and below the mean, respectively. High distress tolerance score indicates lower distress tolerance, in contrast, lower distress tolerance score indicates higher distress tolerance.

Similarly, figure of predicted perceived stress against binge eating behavior was plotted. As shown in **Figure 3**, the higher distress tolerance score, the more prone to binge eating behavior score, regardless of the perceived stress. That is to say, individuals with lower distress tolerance are more likely to engage in binge eating behavior than those with higher distress tolerance.

### Testing for the whole research model

Finally, all the study hypotheses were tested simultaneously using PROCESS macro model 8. The results showed that binge eating behavior was predicted by both perceived stress and life history strategy in Model 6, and life history strategy was predicted by perceived stress in Model 5. Therefore, the relationship between perceived stress and binge eating behavior was partly mediated by life history strategy. In addition, the indirect effect of perceived stress on binge eating behavior via life history strategies was also moderated by distress tolerance (*β* = 0.01, *p* < 0.05. see Model 5 of **Table 4**); meanwhile, the direct effect of perceived stress on binge eating behavior was also moderated by distress tolerance (*β* = 0.01, *p* < 0.05. see Model 6 of **Table 4**). The index of moderated mediation was -0.0006 with a 95% confidence interval (CI) of [-0.0012, -0.0001] (see Model 7 of **Table 4**), the zero value was not included. Therefore, the whole research model was supported.

**Table 4:** Testing for the whole research model.

Model 5 (Life History Strategy)						Model 6 (Binge Eating Behavior)			
Constant	*β*	*st*	*SE*		95%CI	*β*	*t*	*SE*	95%CI
Gender	3.14	4.26^***^	0.74		1.69 ~ 4.59	2.42	8.14^***^	0.30	1.83 ~ 3.00
BMI	0.02	0.16	0.12		-0.22 ~ 0.26	0.19	4.03^***^	0.05	0.10 ~ 0.29
PS	-0.91	-13.25^***^	0.07		-1.04 ~ -0.77	0.17	5.87^***^	0.03	0.11 ~ 0.23
DT	-0.10	-1.95	0.15		-0.20 ~ -0.00	0.15	7.55^***^	0.02	0.11 ~ 0.19
PS×DT	0.01	2.28^*^	0.01		0.00 ~ 0.02	0.01	2.42^*^	0.00	0.00 ~ 0.01
LHS						-0.04	-4.08^***^	0.01	-0.07~-0.02
R^2^	0.20					0.23			
F	65.73					65.31			
Model 7									
Index of moderated moderated mediation	*β*	*SE*		95%CI					
	-0.0006	0.0003		-0.0012~-0.0001					

N=1342,The Beta value are standardized coefficients, thus they can be compared to determine the relative strength of different variables in the model. Each column is a regression model that predicts the criterion at the top of the column.PS, perceived stress, LHS, life history strategy, DT, distress tolerance. *p<0.05, ***p<0.001.

## Discussion

Numerous studies have demonstrated perceived stress is an important trigger of binge eating behavior (6, 15, 45). However, the psychological mechanisms by which perceived stress triggers binge eating behavior are not fully understanded. In the present study, we further addressed this issue from the perspective of evolutionary psychology, focusing on life history strategy and distress tolerance. Firstly, we found a mediating role of life history strategy on the relationship between perceived stress and binge eating behavior. Secondly, when distress tolerance is introduced as a moderator for the perceived stress-life history strategy and perceived stress-binge eating in the model, main interactional effects were also been observed.

There are a number literatures have attempted to explain a variety of psychopathological symptoms within a life history strategy framework (17, 25, 28, 46), including eating disorders (26, 27). However, the evolutionary origin and function of binge eating remains unexplored, and binge eating may have adaptive implications from a life history strategy perspective, especially in situations of high perceived stress.

The term perceived stress refers to a perceived imbalance state between environmental demands and the individual’s capacity to cope with these demands (47). When a person perceives that his abilities are sufficient to cope with the demands of the environment, the perceived stress is low. On the contrary, when he perceives that his abilities are insufficient to cope with the demands of the environment, the perceived stress is high. It has been shown that individuals with binge eating behavior suffer from high standards and expectations, particularly an acute sensitivity to the difficult (perceived) demands of others (16). Therefore, the binge eater is expected to have higher perceived disparity between environmental demands and his capacity to cope with these demands. That is to say, they perceive that they are unable to cope with the demands of their environment and are more likely experience stress overload. In this context, the future is uncertain and hard to predict, it is unlikely that investing will pay off in the future. It is crucial for individuals with binge eating disorder to promptly address excessive psychological stress. Therefore, a fast strategy, focusing more on the present and discounting the future, is more adaptive, where binge eating was used to escape from such emotional distress, particularly when there is no alternative, adaptive emotion-regulation skills (48). The results of the present study supported this hypothesis, finding that life history strategy mediated the relationship between perceived stress and binge eating behavior.

Distress tolerance is another important psychological variable related to coping with stressors. It reflects the perceived and actual capability to withstand aversive physical and psychological states (29, 30). Individuals with low distress tolerance not only have a low threshold for experiencing negative emotions, but also lack emotion regulation skills (49). Previous researchers have found that distress tolerance alone negatively predicted binge eating behavior (50–52), although there are also studies that do not support this view (9, 53). This inconsistency might suggest that distress tolerance may interact with other factors in binge eating. In the present study, we expanded the mechanism of distress tolerance in binge eating. We found that distress tolerance regulates binge eating behavior by interacting with perceived stress through two pathways: (1) the direct effect of perceived stress on the binge eating; (2) the indirect effect of perceived stress on the binge eating through the life history strategy. Therefore, distress tolerance may be an important potential intervention target for binge eating. This was supported by the fact that Dialectical Behavior Therapy (DBT) skills training which include distress tolerance skills reduces binge eating (54).

At the theoretical level, the present study improves our understanding about how perceived stress leads to binge eating behavior in a life history framework. Perceived stress may affect binge eating by fast life history strategy; Distress tolerance moderates both the direct effect of perceived stress on binge eating and its indirect effect through life history strategy.

At the practical level, the present study results suggested that life history-based interventions might effectively relieve stress-induced binge eating behavior. Specifically, developing slow life history strategy could help individuals to reduce their binge eating behavior when encountering similar stressful situations in the future. This is consistent with the idea that understanding eating interventions through an evolutionary lens (55). Further, our results also suggested that distress tolerance skills training might be a potentially effective way to reduce binge eating behavior triggered by perceived stress directly or indirectly by developing slow life history strategy. In fact, previous researches have confirmed that DBT, which includes distress tolerance skills training, significantly reduces binge eating behavior (54, 56). Whether distress tolerance skills training alone also have a similar effect requires further studies in the future.

On the other hand, the present study cannot draw causal conclusions because of its cross-sectional design. Longitudinal studies are still needed to further confirm these conclusions in the future. In addition, although our sample size is relatively large, it is all from one university, which limits the representativeness of the sample. Future research should increase the sampling locations.

## Conclusion

Perceived stress induced binge eating behavior may be mediated by fast life history strategy; Distress tolerance moderates both the direct effect of perceived stress on binge eating and its indirect effect through life history strategy. Therefore, distress tolerance skills training and life history-based interventions might be potentially effective ways to reduce binge eating behavior triggered by perceived stress.

## Data availability statement

The original contributions presented in the study are included in the article/supplementary material. Further inquiries can be directed to the corresponding authors.

## Ethics statement

The studies involving humans were approved by the ethics committee of Guizhou Medical University. The studies were conducted in accordance with the local legislation and institutional requirements. The participants provided their written informed consent to participate in this study.

## Author contributions

XM: Conceptualization, Funding acquisition, Writing – review & editing. JG: Conceptualization, Formal analysis, Investigation, Writing – original draft. YL: Writing – review & editing. BX: Writing – review & editing. CL: Writing – review & editing, Conceptualization, Funding acquisition, Writing – original draft.
